# High-Power DFB Diode Laser-Based CO-QEPAS Sensor: Optimization and Performance

**DOI:** 10.3390/s18010122

**Published:** 2018-01-04

**Authors:** Yufei Ma, Yao Tong, Ying He, Xin Yu, Frank K. Tittel

**Affiliations:** 1 National Key Laboratory of Science and Technology on Tunable Laser, Harbin Institute of Technology, Harbin 150001, China; tongyao9505@163.com (Y.T.); hearkenyi@hit.edu.cn (Y.H.); yuxin0306@hit.edu.cn (X.Y.); 2 Department of Electrical and Computer Engineering, Rice University, 6100 Main Street, Houston, TX 77005, USA

**Keywords:** QEPAS, carbon monoxide, high power diode laser, gas sensor

## Abstract

A highly sensitive carbon monoxide (CO) trace gas sensor based on quartz-enhanced photoacoustic spectroscopy (QEPAS) was demonstrated. A high-power distributed feedback (DFB), continuous wave (CW) 2.33 μm diode laser with an 8.8 mW output power was used as the QEPAS excitation source. By optimizing the modulation depth and adding an optimum micro-resonator, compared to a bare quartz tuning fork (QTF), a 10-fold enhancement of the CO-QEPAS signal amplitude was achieved. When water vapor acting as a vibrational transfer catalyst was added to the target gas, the signal was further increased by a factor of ~7. A minimum detection limit (MDL) of 11.2 ppm and a calculated normalized noise equivalent absorption (NNEA) coefficient of 1.8 × 10^−5^ cm^−1^W/√Hz were obtained for the reported CO-QEPAS sensor.

## 1. Introduction

Carbon monoxide (CO) is an air pollutant that is produced by incomplete production combustion activities, such as combustion of natural gas, fossil fuels and other carbon-containing fuels for power generation, petrochemical refining and vehicle or truck transportation. CO in the atmosphere can react with hydroxyl (OH) to aggravate global warming indirectly [1]. CO is an important target molecule in hydrocarbon fuel systems, and can be regarded as indicating combustion efficiency [2]. In addition, the presence of CO in exhaled breath is associated with human diseases at concentration levels of ppm [3,4,5]. Furthermore, excessive exposure to CO can cause deprivation of oxygen in human tissue [6]. Therefore, sensitive detection of CO concentration levels has an important significance.

Quartz-enhanced photoacoustic spectroscopy (QEPAS) technique was first reported in 2002 as a modification of conventional photoacoustic spectroscopy [7]. QEPAS eliminates the limitation of a gas cell caused by sound resonance conditions. The quartz tuning fork (QTF) can be placed in the near-field area of the excitation laser beam [8,9,10,11]. Therefore, the sealing of the gas is not necessary in this technique, and is only used to separate the gas sample from the surroundings in order to control its pressure. An important feature of a QTF is its low price, small volume, and high quality factor (Q value ~ 10,000 at a standard atmospheric pressure) [12,13,14,15]. Usually in QEPAS a QTF with resonant frequency *f*_0_ of 32,768 Hz acts as an acoustic transducer. Therefore, the corresponding energy accumulation time (t = Q/*f*_0_) is ~300 ms which is significantly longer than any photoacoustic cell used in traditional microphone based photoacoustic spectroscopy. Furthermore, a QTF has very good immunity to ambient noise due to its acoustic quadrupole geometry and a narrow response frequency band (~4 Hz) [16]. QEPAS has been successfully applied to the detection of numerous trace gases [17,18,19,20,21,22,23] due to its advantages of high sensitivity, selectivity, and compactness, as well as its fast temporal response.

A QEPAS-based sensor for CO detection employing a 4.6 μm quantum cascade laser (QCL) as the excitation source was reported in Refs. [24,25]. Although the strongest absorption line could be targeted and a high excitation power can be achieved when employing a QCL, such CO-QEPAS sensor systems suffer from high cost, high power consumption and size. Compared with QCLs, diode lasers with emission wavelength of <3 μm have several advantages, such as compactness and significantly lower cost. The diode laser can access first overtone absorption band of CO, located at 2.3 μm, and diode laser-based CO-QEPAS sensors have been reported previously in Refs. [26,27].

The QEPAS signal amplitude *S* is given by Equation (1) [17]:

(1) $$S\propto \frac{\alpha PQ}{f_{0}}$$

where *α* is the absorption coefficient, *P* is the optical power, *Q* is the Q-factor of QTF, *f*_0_ is the QTF resonance frequency. An important feature of QEPAS is that the performance of QEPAS-based sensors can be improved when the excitation laser power is increased [28], since QEPAS detection sensitivity scales linearly with excitation laser power *P* (see Equation (1)). However, to date, commercially available 2.3 μm diode lasers have a maximum output power of ~2 mW, which limits the CO-QEPAS sensor performance. 

In this paper, a sensitive QEPAS-based CO trace gas sensor was demonstrated. A high-power distributed feedback (DFB) diode laser with a fiber pigtail output power of ~9 mW was used as the excitation source. The laser wavelength modulation depth and the length of micro-resonator were optimized. Furthermore, enhancement of the CO-QEPAS signal was realized by the addition of water vapor to improve the CO vibrational-translational relaxation rate.

## 2. Experiment Setup

**Figure 1:**
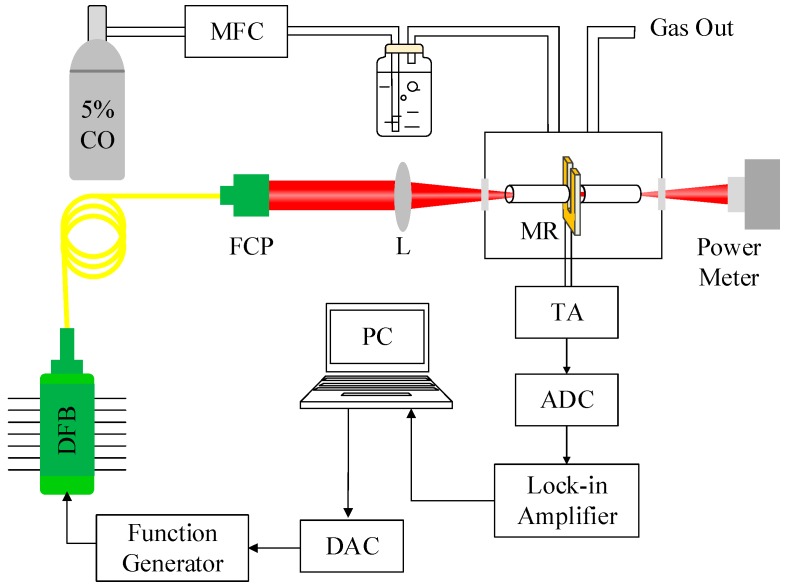
Schematic of a high-power DFB, CW diode laser-based CO-QEPAS sensor platform.

A schematic of the QEPAS-based CO sensor platform is shown in Figure 1. As an excitation source, a 2.3 μm fiber-coupled, DFB, continuous-wave (CW) diode laser (Model #: KELD1G5BAAH, NEL Corp., Kawasaki City, Japan) in a 14-pin butterfly package that included a thermoelectric controller (TEC) operating at 19 °C was employed. The current of the DFB-CW diode laser was modulated at half the resonance frequency (*f* = *f*_0_/2 ≈ 16.3 kHz) of the QTF. The laser beam was collimated and focused between the QTF prongs by using a fiber collimation package (focal length: 11 mm, Model #: F021APC-2000, Thorlabs, Newton, NJ, USA) and a plano-convex lens (L) with a focal length of 40 mm. A transimpedance amplifier (TA) with a resistance of 10 MΩ was used to convert the piezoelectric current to voltage. The voltage signal was used to demodulate the second harmonics (2*f*) signal. Two stainless steel tubes formed an acoustic micro-resonator (MR), which improved the detection sensitivity of the QEPAS system. The sensor system was processed by a laptop computer (PC) using LabVIEW software.

**Figure 2:**
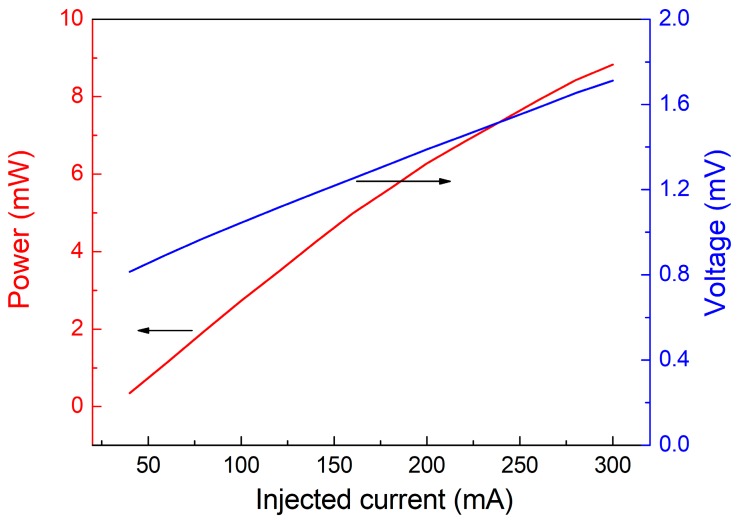
2.3 μm diode laser output performance.

From Figure 2, we can see that the maximum optical power emitted by the 2.3 μm fiber-coupled, DFB-CW diode laser operating at a temperature of 19 °C and an injection current of 300 mA was ~8.8 mW.

**Figure 3:**
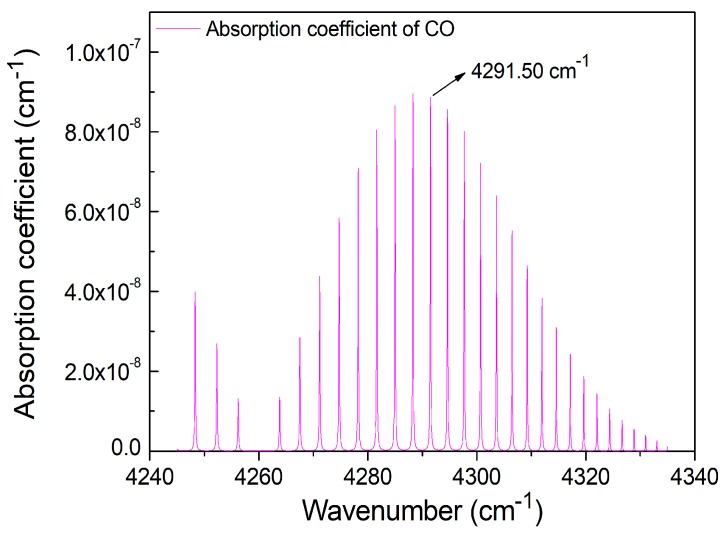
Absorption lines for CO molecules in the 2.3 μm first overtone absorption band based on the HITRAN 2012 database.

The 200 ppb CO absorption lines in the 2.3 μm first-overtone absorption band at a temperature of 296 K and a standard atmospheric pressure, according to the HITRAN 2012 database [29], are shown in Figure 3. This simulation shows that a line located at 2330.19 nm (4291.50 cm^−1^) with an absorbance coefficient of 8.85 × 10^−8^ cm^−1^ is one of the strongest lines, at ~2.3 μm. The DFB-CW diode laser wavelength can be tuned to cover this absorption line by changing the laser injection current at a constant TEC temperature of 19 °C.

## 3. Results and Discussion

The QEPAS sensor performance can be significantly improved when an acoustic micro-resonator (MR) is added. There are two main kinds of micro-resonators, an on-beam and an off-beam MR [30,31,32]. Compared to an off-beam configuration, the on-beam has the advantage of a stronger acoustic coupling efficiency. Therefore, in this research, an on-beam architecture was chosen. The optimal length of MR range is λ_s_/4~λ_s_/2, where λ_s_ is the sound wavelength. The calculated optimal length of the MR is 2.6~5.2 mm, based on the speed of sound, which is 340 m/s in 5% CO:N_2_. In this experiment, the lengths of the stainless tubes were 3 mm, 4 mm, 5 mm or 5.5 mm. The inner diameter of the stainless tubes was 0.5 mm, and the gap between the QTF and MR tubes was 25 μm. The optimal distance from QTF tips to the axis of MR was chosen to be 0.7 mm [11].

**Figure 4:**
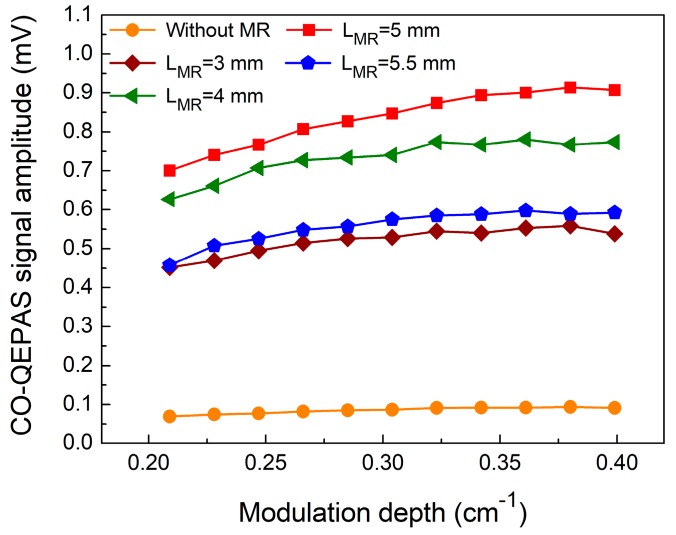
CO-QEPAS signal amplitude as a function of modulation depth.

**Figure 5:**
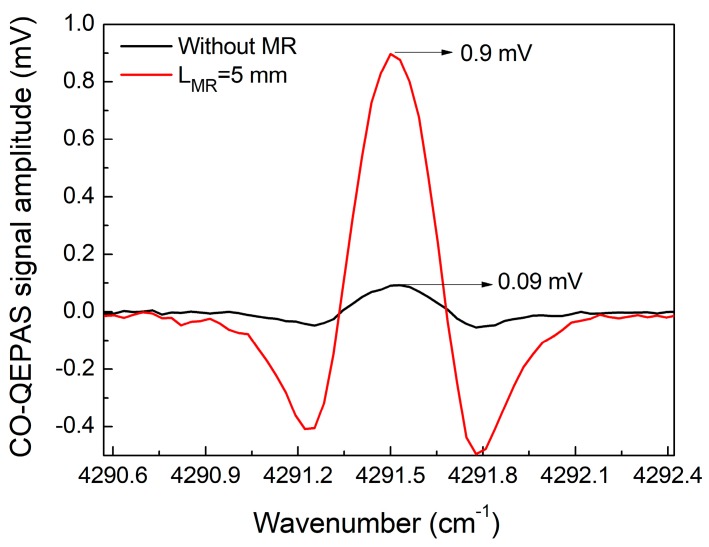
QEPAS signal amplitude without MR and MR with a length (L_MR_) = 5 mm.

Figure 4 shows the QEPAS signal amplitude as a function of laser wavelength modulation depth for 4 different MRs for a 5% CO:N_2_ gas mixture. The QEPAS signal amplitude increased with the modulation depth. However, when the modulation depth was >0.38 cm^−1^, the signal amplitude did not change. It can be seen that the CO-QEPAS signal improved when the MRs were added. A maximum signal enhancement of 10 times was obtained when a MR with a length (L_MR_) of 5 mm was used. For this condition, the QEPAS system had the strongest acoustic coupling. The measured 2*f* QEPAS signal with a modulation depth of 0.38 cm^−1^ and L_MR_ = 5 mm is depicted in Figure 5.

**Figure 6:**
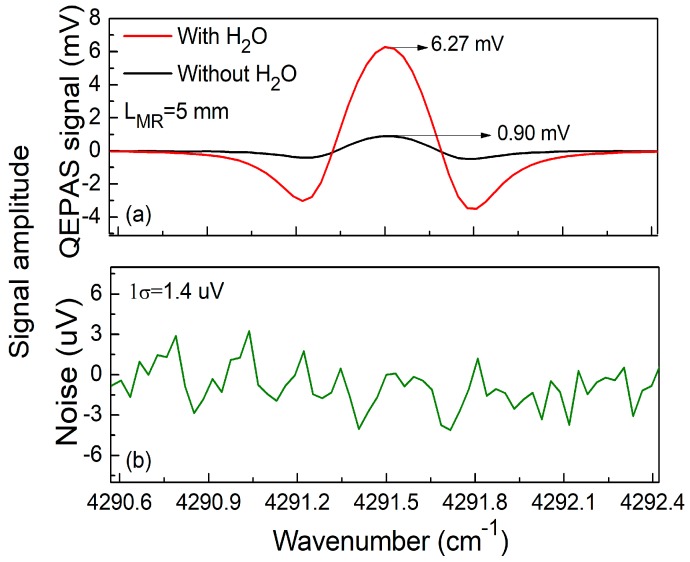
Signal amplitude. (**a**) QEPAS signal based on a 5 mm MR with and without H_2_O; (**b**) Pure N_2_ for a noise background determination.

Enhancement of the CO-QEPAS signal was realized by the addition of water vapor to improve the CO vibrational-translational relaxation rates. The addition of water vapor with a concentration of 1.01% in the gas mixture resulted in a signal improvement of ~7 fold, as shown in Figure 6a. This confirmed that water vapor is an efficient catalyst for the vibrational-relaxation energy reactions in the gas phase. Figure 6b depicts the background signal measured when the QEPAS sensor cell was flushed with high-purity nitrogen (N_2_). The 1σ background signal was 1.4 μV. QEPAS background noise is limited by the fundamental Johnson thermal noise of the QTF. Based on the data depicted in Figure 6, the minimum detection limit (MDL) was 11.2 ppm for a 1 sec time constant of the lock-in amplifier, which is significantly better than 43.3 ppm, as reported in Ref. [27]. The calculated normalized noise equivalent absorption (NNEA) coefficient for the reported CO-QEPAS sensor was 1.8 × 10^−5^ cm^−1^W/√Hz.

The above measurements were carried out at room temperature (296 K) and atmospheric pressure (1 atm). Wavelength modulation spectroscopy (WMS) with 2nd harmonic detection was utilized for concentration measurements [33,34] in this paper. The absorption coefficient in WMS can be expanded into a Fourier series. *H_n_*(*v*) is the nth Fourier component of the modulated absorption coefficient, and is expressed as Equation (2) [35]:

(2) $$H_{n}(\nu_{0},\nu_{a})=\frac{2^{1-n}I_{0}L}{n!}\nu_{a}^{n}{\left[\frac{d^{n}\alpha (\nu )}{d\nu^{n}}\right]}_{\nu =\nu_{0}}=\frac{2^{1-n}I_{0}NLS(T)}{n!}\nu_{a}^{n}{\left[\frac{d^{n}g(\nu )}{d\nu^{n}}\right]}_{\nu =\nu_{0}},\;n\geq 1$$

where *I*_0_ is the laser intensity, *N* is the molecular density, *L* is the absorption pass length, *S*(*T*) is the absorption line intensity, *g*(*v*) is the line shape function.

(3) $$P_{2f}\propto H_{2}(\nu_{0},\nu_{a})=\frac{2^{-1}I_{0}NLS(T)}{n!}\nu_{a}^{2}{\left[\frac{d^{2}g_{V}(\nu )}{d\nu^{2}}\right]}_{\nu =\nu_{0}}$$

**Figure 7:**
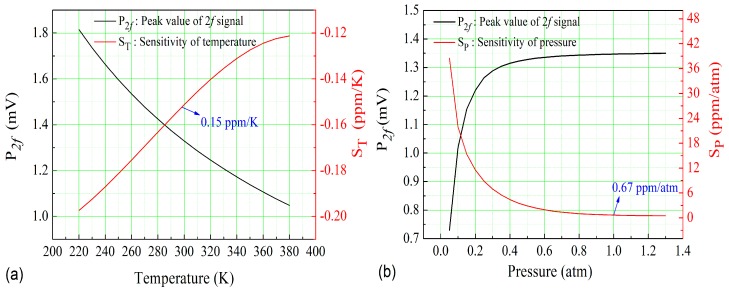
(**a**) 2*f* signal intensity and temperature sensitivity at different temperatures; (**b**) 2*f* signal intensity and pressure sensitivity at different pressures.

The second harmonic acoustic signal P_2*f*_ of QEPAS is proportional to *H*_2_(*v*), as shown in Equation (3). The 2*f* signal intensities at different temperatures and pressures were calculated according to the above equation, and are shown in Figure 7. The temperature sensitivity and pressure sensitivity for the concentration retrieval were calculated based on the derivation of 2*f* signal, and are also depicted in Figure 7a,b. It can be seen that the temperature sensitivity and pressure sensitivity are 0.15 ppm/K and 0.67 ppm/atm, respectively, at 296 K and 1 atm, which means that, under the laboratory conditions, the CO-QEPAS sensor was not insensitive to the environmental variables.

## 4. Conclusions

In conclusion, a sensitive CO-QEPAS sensor based on a high-power DFB-CW diode laser was demonstrated. Due to the fact that QEPAS detection sensitivity scales linearly with excitation laser power, the 8.8 mW diode laser output power was advantageous for improving sensor performance. The laser wavelength modulation depth was optimized. Different micro-resonators with lengths of 3 mm, 4 mm, 5 mm and 5.5 mm were added to both sides of the QTF to form an acoustic resonant cavity to improve the signal amplitude. Further enhancement of the CO-QEPAS signal was realized by the addition of water vapor with a 1.01% concentration to improve the CO vibrational-translational relaxation rates. Finally, an excellent MDL of 11.2 ppm and a calculated normalized noise equivalent absorption (NNEA) coefficient of 1.8 × 10^−5^ cm^−1^W/√Hz were obtained. With a CO detection sensitivity of 10 ppm concentration levels, the reported CO-QEPAS-based sensor is suitable for applications in environmental monitoring, combustion science and other applications.
